# Host sweet preference modulates the salivary microbiome and its divergent associations with plaque-associated and non-plaque-related oral diseases

**DOI:** 10.3389/fmicb.2025.1732083

**Published:** 2025-12-05

**Authors:** Zhenzhen Li, Xiao Peng, Qi Wang, Liangying Guo, Shanshan Liu, Li Xu

**Affiliations:** 1 Department of Stomatology, The First Affiliated Hospital of Bengbu Medical University, Bengbu, China; 2 Cuiying Biomedical Research Center, Lanzhou University Second Hospital, Lanzhou, China

**Keywords:** oral microbiome, sweet consumption frequency, dental caries, dental fluorosis, salivary

## Abstract

**Background:**

Microorganisms play a critical role in the progression of oral diseases. However, it remains unclear whether the frequency of sweet consumption influences the salivary microbiota in both plaque-associated and non-plaque-related oral diseases.

**Methods:**

Based on salivary microbiome analysis, unstimulated saliva samples were collected from university students aged 17–20, including healthy controls (HC), dental caries (DC), and dental fluorosis (DF) groups, under different sweet consumption frequencies. Microbiota potentially critical in disease development were identified.

**Results:**

No significant differences in α- and β-diversity were observed among the three groups. However, distinct microbial structures at the genus and species levels were evident under different sweet consumption conditions. Under high sweet consumption, the caries group exhibited enrichment of microbiota closely associated with sugar metabolism and acid production (e.g., *Streptococcus, Rothia*), while *Ralstonia* was significantly enriched in the caries group, suggesting its potential role in high-sweet-induced caries development. Under low sweet consumption, the healthy control group showed enrichment of taxa such as *Stenotrophomonas*, potentially linked to ecological stability, whereas the dental fluorosis group demonstrated significant enrichment of *Fastidiosipila*, reflecting specific fluoride-induced selective pressure on the microbiome. This study indicates that although sweet consumption frequency did not significantly alter overall microbial diversity, it reshaped the oral microbiota structure in a disease-specific context. The caries group was more prone to developing a cariogenic microbial profile under high-sugar conditions, while the fluorosis group exhibited unique ecological adaptive characteristics.

## Introduction

The distinctive anatomical structure of soft and hard tissues in the oral cavity (including teeth, gingival sulcus, attached gingiva, tongue surface, buccal mucosa, and palate) provides a variety of ecological niches for microbial communities (Baker et al., 2023). Over 700 species of oral microorganisms have currently been identified, which can either float in saliva as planktonic forms or adhere to oral surfaces to form dental plaque biofilms (Xian et al., 2018). Under healthy conditions, these microorganisms maintain a dynamic balance with the host, collectively contributing to oral health (Baker et al., 2023). However, when this ecological balance is disturbed, causing dysbiosis, it may lead to the onset of several oral diseases. These diseases are traditionally classified into two categories based on their etiology: plaque-related oral diseases, which are directly associated with the pathogenic transformation of plaque microorganisms (Sbordone and Bortolaia, 2003) and non-plaque-related oral diseases, whose causes are typically linked to environmental factors, systemic factors, immune status, or trauma (Hung et al., 2023; Reichart and Nguyen, 2008).

Among oral diseases, dental caries is the most common plaque-related diseases. According to data from the 2017 Lancet report, approximately 2.3 billion people are affected by permanent tooth caries, making it the most prevalent disease globally and the third highest in incidence among 354 diseases assessed (James et al., 2018). If dental caries is not treated promptly, it can lead to severe complications, including pulpitis, apical periodontitis, and potentially life-threatening infectious endocarditis (Pitts et al., 2017). Fluoride is often referred to as a “double-edged sword.” This is because an appropriate intake of fluoride can effectively prevent dental caries, while excessive fluoride intake may cause harmful effects on different tissues of the body, such as teeth, bones, and soft tissues (Pramanik and Saha, 2017). Dental fluorosis is a chronic fluorine toxicity manifestation caused by excessive fluoride intake during tooth development, characterized by abnormalities in enamel structure and morphology. Severe cases may be associated with tooth wear, further affecting oral function and social interactions (Hung et al., 2023; Arheiam et al., 2022; Ghosh et al., 2024).

Recent studies have revealed that the oral microbiome is strongly dependent on various factors, including environmental influences, systemic conditions, and immune status, with diet playing a crucial role (Shen et al., 2024). Recent studies have further revealed the specific effects of dietary sugars on the oral microbiome: high sugar content beverages (>3 cans/week) and high sweet treat consumption leads to changes in the overall bacterial community structure and also significantly reduces the species richness of the oral microbiota (Fan et al., 2024; Lommi et al., 2022). This is primarily characterized by the over-proliferation of acid-producing bacteria in the saliva of individuals with high sugar intake, such as the significant increase in the abundance of *Bifidobacteriaceae*, *Lactobacillus rhamnosus*, as well as *Streptococcus*, *Prevotella*, *Veillonella*, and *Selenomonas*, which disrupts the microbial ecological balance and promotes the onset and development of caries. Based on this evidence, the World Health Organization recommends limiting free sugar intake to less than 10% of total energy intake to reduce the risk of caries (Moynihan and Kelly, 2013). However, current research has notable limitations, with nearly all focus placed on plaque-related diseases. Could the intake of sweet foods affect the development of non-plaque-related oral diseases by altering the overall state of the oral microenvironment (such as pH, salivary flow, and immune components)? Does the frequency of sweet food consumption have a consistent impact on the oral salivary microbiota in both plaque-related and non-plaque-related diseases? These issues have rarely been addressed so far.

In our previous large-scale population cohort study, we found significant differences in the frequency of sweet food intake between patients with typical plaque-related disease-dental caries and non-plaque-related disease-dental fluorosis. In this regard, the present study uses a cross-sectional design, integrating high-throughput sequencing technology and bioinformatics tools to systematically compare sweet food consumption and the differences in salivary microbiome composition among dental caries patients, dental fluorosis patients, and caries-free individuals. The study aims to deepen the understanding of how dietary habits shape the salivary microbiome and their long-term impact on oral health, particularly in the context of different oral health statuses. Given the continuing rise in global oral disease incidence, the findings of this study are expected to provide scientific evidence for the development of more targeted dietary guidelines and preventive strategies, offering important insights for oral health management in younger populations.

## Materials and methods

### Study population

This study was conducted on the campus of Bengbu Medical University, with the research protocol approved by the University’s Ethics Committee. All participants provided their written informed consent before their participation. The sample size was estimated based on data from the Fourth National Oral Health Epidemiology Survey of China, using the formula *N* = (*Z*_*α*/2_/*δ*)^2^ * *P*(1 − *P*) to ensure sufficient statistical power for estimating the prevalence of the target diseases. For the dental caries survey, the parameters were set as *α* = 0.05, *Z*_*α*/2_ = 1.96, *δ* = 0.05, and *p* = 44.42% (derived from the prevalence among 15-year-olds) (Cheng et al., 2020), resulting in a minimum required sample size of 378 participants. For the dental fluorosis survey, *P* was set to 13.4% (Zhou et al., 2018), while other parameters remained unchanged, yielding a minimum sample size of 178 participants. A total of 957 college students were recruited. The study population is the same as that described in our previously published article (Liu et al., 2023), though the current research addresses a distinct scientific objective. A final analytical sample of 299 participants was selected for microbiome analysis. This cohort comprised 100 healthy controls (HC), 99 with dental fluorosis but no caries (DF), and 100 with dental caries (DC). Based on the frequency of sweets consumption, each group was stratified into high-frequency (>1 time/day) and low-frequency (≤1 time/day) subgroups, yielding the following groups: HC-L, HC-H, DF-L, DF-H, DC-L, and DC-H. All participants met the following inclusion criteria: no history of recurrent oral ulcers, pulp or periodontal diseases, or other oral pathologies; absence of systemic or genetic disorders; and no use of antibiotics or exposure to tobacco products within the past 6 months.

The oral health examination was conducted in accordance with the World Health Organization (WHO) Oral Health Surveys Methods (Ben Yahya, 2024). Standardized instruments, including plane mirrors, tweezers and CPI probes, were applied to assess dental caries (documented by ICDAS index) and dental fluorosis (classified by Dean’s index). To ensure data robustness, all examinations were carried out by dentists from the Department of Stomatology, First Affiliated Hospital of Bengbu Medical University. Prior to the survey, all examiners received uniform training. Calibration for diagnosing caries and fluorosis showed Kappa values exceeding 0.8, indicating strong inter-examiner reliability. A questionnaire was then administered to the participants to gather information on their parental education level, use of fluoride toothpaste, toothbrushing and flossing frequency, and sweet food intake frequency.

### Saliva collection

Saliva samples were collected from all participants before the clinical assessment. Participants were instructed to refrain from eating or drinking for 2 h prior to sampling and to rinse their mouths with water before collection. Unstimulated whole saliva, free of sputum, was collected by placing a 5 mL centrifuge tube under the lower lip until at least 3 mL of saliva was obtained. One milliliter of each sample was reserved for microbiome analysis. All samples were stored at −80 °C within 4 h of collection for subsequent processing.

### Saliva DNA extraction, 16S rRNA sequencing analysis

Genomic DNA was extracted using the CTAB or SDS method. After assessing purity and concentration by agarose gel electrophoresis, appropriate aliquots were transferred into centrifuge tubes and diluted with sterile water to a final concentration of 1 ng/μL for use as PCR templates. The 16S rRNA gene harbors nine hypervariable regions (V1–V9). As reported by Digvijay Verma and colleagues, in NGS-based oral microbiome studies, the V1–V2 and V3–V4 regions are commonly targeted for amplification owing to their pronounced sequence variability and rich taxonomic information. These regions provide robust resolution for distinguishing bacterial groups and have been shown to be highly representative and reliable in revealing the phylum-level architecture of the oral microbiome (Verma et al., 2018). Guided by this evidence, we selected the V3–V4 region as the target for amplification in this study. The V3–V4 region was amplified by PCR using individual-specific barcoded primers, 341F (5′-CCTAYGGGRBGCASCAG-3′) and 806R (5′-GGACTACNNGGGTATCTAAT-3′), with primer design and selection guided by reference (He et al., 2022).

Libraries were constructed with the TruSeq® DNA PCR-Free Sample Preparation Kit (Illumina, USA) and paired-end sequencing (2 × 250 bp) was performed on the Illumina NovaSeq platform. Initial processing removed barcodes and primers with Perl v5.0, after which paired-end reads were merged into complete sequences using FLASH v1.2.7^1^ (Magoč and Salzberg, 2011). Quality control was conducted with fastp,^2^ producing high-quality clean reads, which were further processed with VSEARCH^3^ (Yu et al., 2022) to eliminate chimeras, resulting in valid reads for subsequent analyses. Using QIIME2, denoising was performed via the DADA2 module, with sequences of abundance <5 excluded, thereby producing ASVs and their corresponding feature tables. Taxonomic classification of ASVs was achieved via the classify-sklearn module in QIIME2 by aligning sequences against reference databases (Davis et al., 2019). Taxonomic analysis was conducted mainly with the eHOMD database (Escapa et al., 2018) and the NCBI Taxonomy database.^4^

### Sequencing depth and sequencing quality control

Yielding an average of 84,624 reads per sample across 299 samples. After quality control, an average of 68,515 high-quality reads per sample were retained. We further evaluated the impact of rarefaction thresholds on the representation of community structure and confirmed that a depth of 36,365 reads per sample was sufficient to capture the majority of microbial diversity in most samples. All downstream analyses were conducted using this rarefied dataset to ensure robustness and reliability of the results.

During the experimental process, extracted deionized water was used as a negative control to identify potential contaminating sequences derived from reagents or the environment. Following sequencing, any ASVs detected in the negative controls were removed from the actual samples to eliminate low-frequency signals that may represent technical noise or incidental contamination.

### Statistics

Analyses of microbial communities were conducted using the QIIME2 platform. α-diversity was estimated using the Chao1 index, Observed ASVs and Shannon index (Li et al., 2013), and statistical differences among groups were tested by Wilcoxon test. Microbial community composition (β-diversity) was computed from Bray–Curtis distance matrices, with principal coordinate analysis (PCoA) performed and visualized in R. β-diversity was compared using Permutational Multivariate Analysis of Variance (PERMANOVA), implemented with the vegan package (version 2.5–7) in R. Differential taxa were identified using a combination of LEfSe analysis (LDA score >3.0) and statistical testing (Wilcoxon test) across phylum, genus, and species levels, with significance set at false discovery rate (FDR) < 0.05 and fold-change (FC) > 2 or < 0.05. Venn diagram was used to compare the differential microorganisms at the genus and species level among different groups of samples. Receiver operating characteristic (ROC) curves were employed to assess the efficacy of microbial markers in distinguishing among different groups.

**Figure 1:**
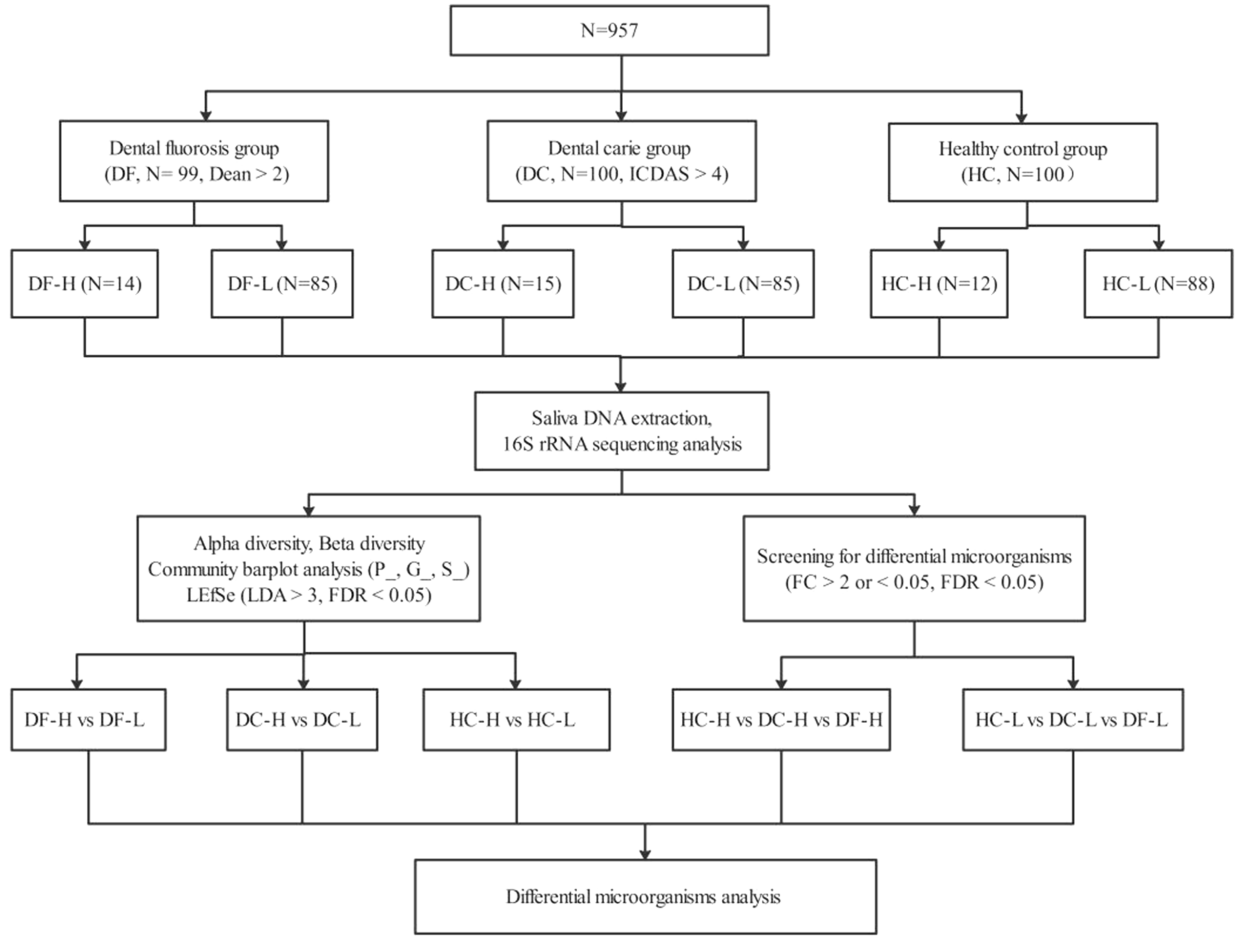
The workflow of the current study.

An LDA score threshold of 3.0 (corresponding to a 1,000-fold effect size) was applied, consistent with common practice in microbiome research to balance sensitivity and specificity when identifying biomarkers with large effect sizes and clear biological relevance. To evaluate LDA assumptions, the analysis was performed using normalized relative abundance data, and the intrinsic log-transformation in LEfSe helped approximate normality. Although microbial data rarely satisfy all theoretical assumptions of LDA, we cross-validated the LEfSe results using non-parametric tests (Wilcoxon test with FDR correction) and observed strong concordance between the methods. This agreement reinforces the robustness of the identified biomarkers and indicates that our findings are not dependent on the assumptions of any single method (see Figure 1).

## Results

### Comparison of salivary microbes between HC-L and HC-H

**Figure 2:**
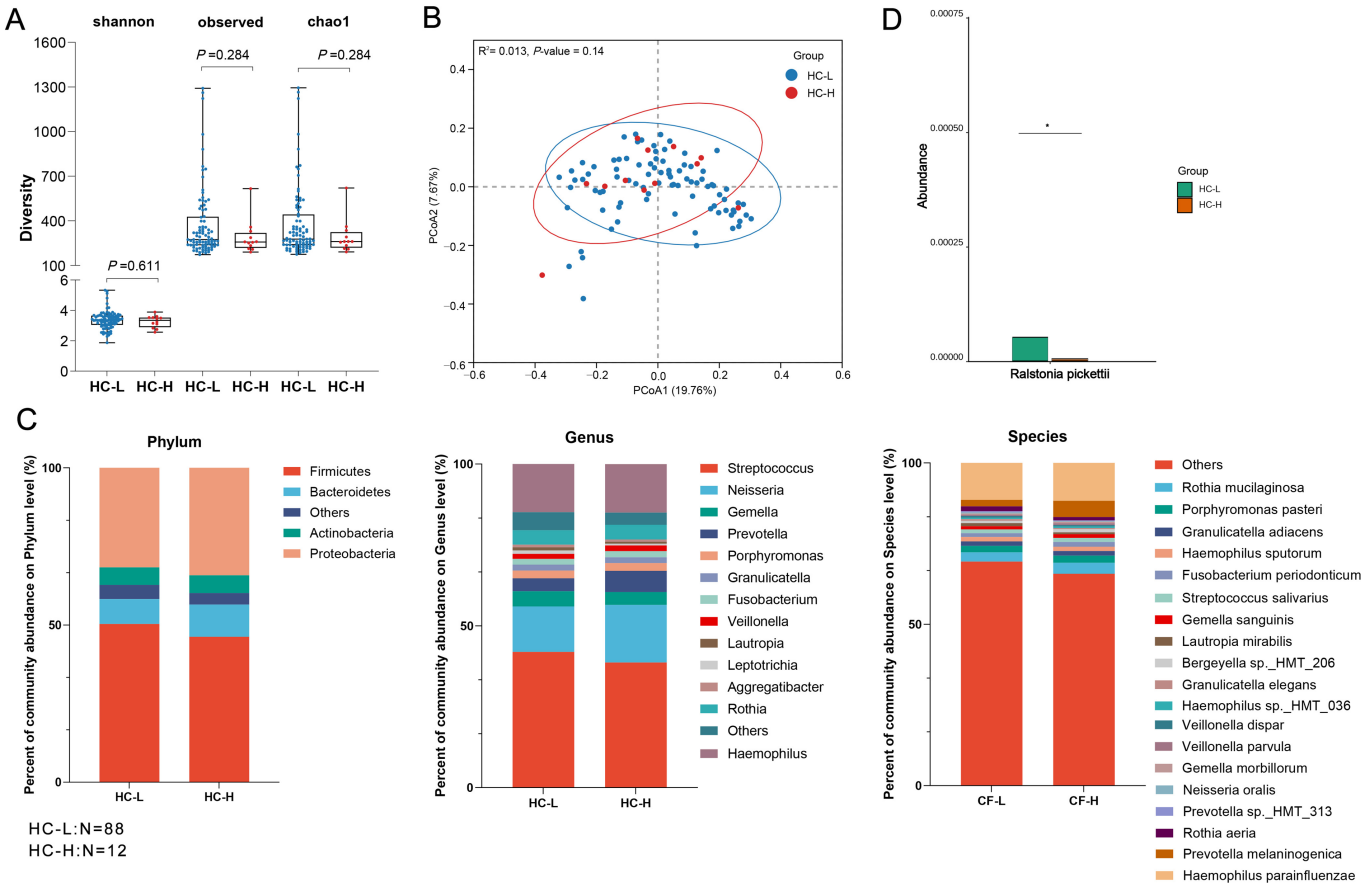
The microbiome analysis between HC-L and HC-H. **(A)** Alpha diversity comparison, showing no significant differences in diversity between the two groups (*p* > 0.05). **(B)** PCoA (Principal Coordinates Analysis) plot based on Bray–Curtis distances, illustrating the microbial community structure between the two groups. The *R*^2^ value is 0.013, and the *p*-value is 0.014, indicating a weak but statistically significant separation between the groups. **(C)** Average relative proportions of the main phyla, genera, and species in the two groups. Bar charts represent the relative abundance of microbial taxa at different taxonomic levels. **(D)** Bar chart depicting the comparison of a specific microbial taxa between the two groups, identified by LEfSe analysis (LDA > 3). The comparison shows a statistically significant difference in the abundance of this microorganism between the groups (*p* < 0.05). Alpha diversity was assessed using Wilcoxon test, PCoA analysis was conducted with Bray–Curtis distances. HC-H (*N* = 12): the high sweet consumption frequency in the healthy control group; HC-L (*N* = 88): the low sweet consumption frequency in the healthy control group.

Based on the microbiome analysis results, ASV-level analysis was performed on the α-diversity indices (such as Shannon index, Chao1 index, observed_ASVs) for high and low sweet consumption frequency groups in the healthy population (HC) (Figure 2A). The results revealed that the *p* > 0.05, suggesting no significant differences in α-diversity metrics such as richness and evenness between the high and low sweet consumption frequency groups in the healthy population. Based on Bray–Curtis distance, PCoA was used to reduce the complex microbiome structure to a two-dimensional scatter plot. The PERMANOVA method was used to calculate the statistical indicators *R*^2^ and *p*-value to verify whether the observed separation trend was statistically significant. It was observed that the HC group did not exhibit a clear separation trend on PC1 (explaining 19.76% of the variance) and PC2 (explaining 7.67% of the variance) (*R*^2^ = 0.013, *p* = 0.14), suggesting that sweet consumption frequency does not significantly change the overall composition of the oral microbiome (Figure 2B). We further selected high-abundance bacteria specific to the healthy population and compared them at the phylum, genus, species levels (Top 5—Top 20 abundance) (Figure 2C). The results showed that at the phylum level, there were no significant differences between the two groups in the top five dominant phyla. At the genus and species levels, the proportion of *Leptotrichia* (a genus of *Fusobacteria*) and *Lautropia mirabilis* was higher in the low sweet consumption group (HC-L) than in the high sweet consumption group (HC-H), suggesting they may be the dominant bacteria in the low sweet group. The LEfSe algorithm was used to further confirm the differential microbiomes between the two groups, and the results showed that *Desulfobulbus* sp.*_HMT_041* (*Desulfobulbus HMT_041*) had a higher LDA score in the low sweet consumption group (HC-L), suggesting its significant representativeness in this group.

### Comparison of salivary microbes between DC-L and DC-H

**Figure 3:**
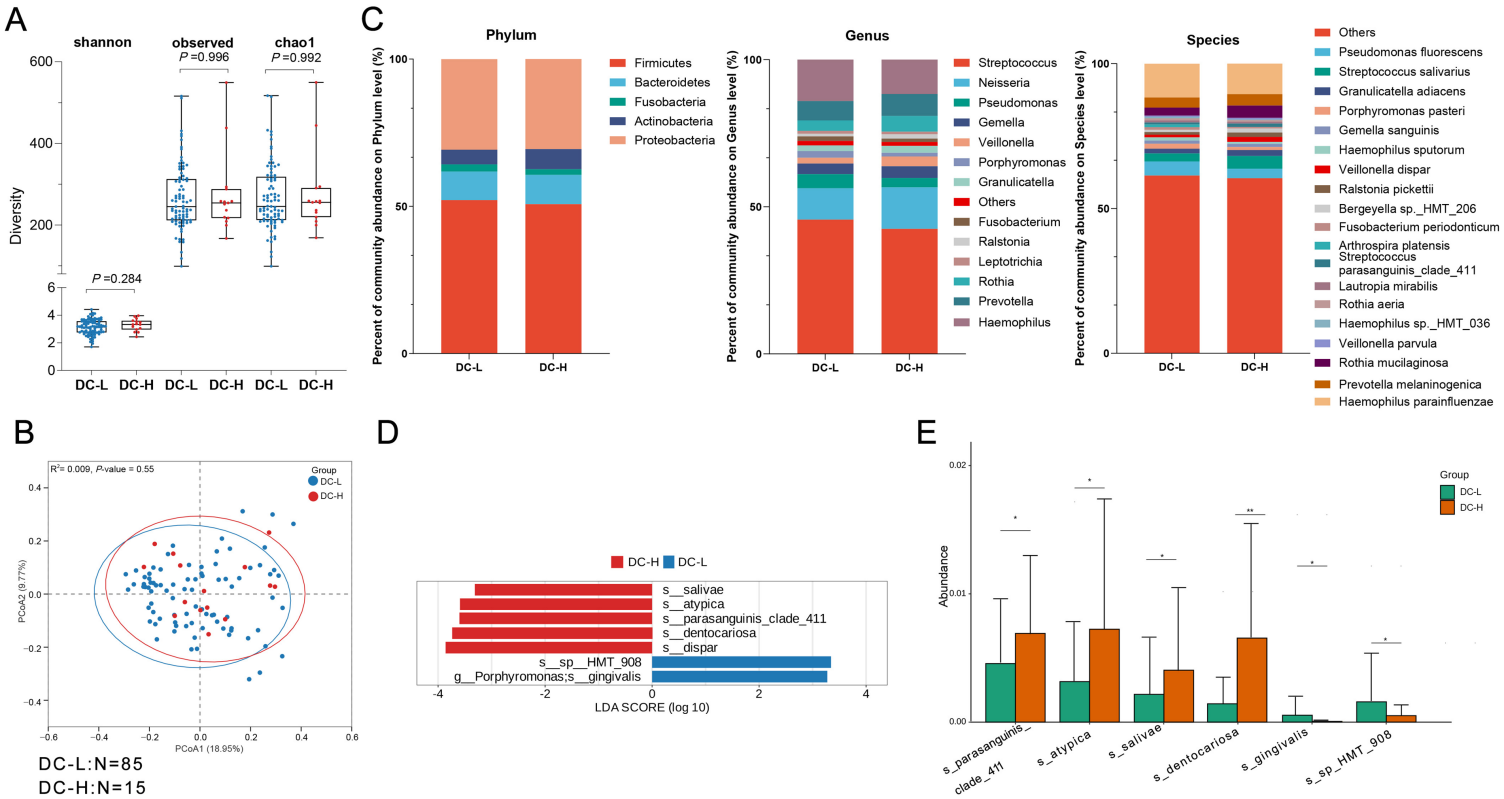
Comparison of oral microbiota diversity and composition between DC-L and DC-H groups. **(A)** Alpha diversity comparison showing no significant differences between the DC-L and DC-H groups for Shannon, Observed, and Chao1 indices (*p* > 0.05, Wilcoxon test). **(B)** PCoA (Principal Coordinates Analysis) plot based on Bray–Curtis distances, illustrating the microbial community structure between the two groups. Each point represents a sample, and colors differentiate the groups. The *R*^2^ value is 0.009, and the *p*-value is 0.55, indicating no significant separation between the groups. **(C)** Average relative proportions of the main phyla, genera, and species in the two groups. Bar charts show the relative abundance of microbial taxa at different taxonomic levels. **(D)** LEfSe analysis (LDA > 3) identifying key bacterial taxa with significant differences in abundance (from genus to species level) between the DC-L and DC-H groups. Only significant taxa with LDA scores greater than 3 are displayed. **(E)** Bar chart showing the comparison of a specific microbial taxon identified by LEfSe analysis (LDA > 3) between the two groups, illustrating the significant difference in microbial abundance between DC-L and DC-H. DC-H (*N* = 15): the high sweet consumption frequency in the dental caries group; DC-L (*N* = 85): The low sweet consumption frequency in the dental caries group.

Next, we compared the differences in salivary microbiome composition between the high sweet consumption frequency (DC-H) and low sweet consumption frequency (DC-L) groups in the caries group (DC). The results indicated that no significant differences were observed between DC-L and DC-H in both α-diversity (ASV level) and β-diversity analysis (*R*^2^ = 0.009, *p* = 0.55), implying that variations in sweet consumption frequency do not significantly alter the internal diversity or overall structure of the oral microbiome in the caries group (Figures 3A,B). Further comparison of the high-abundance microbiota (Top 5—Top 20, covering phylum, genus, species levels) between the two groups (Figure 3C) showed that there were no significant differences in high-abundance bacteria at the phylum level. At the genus and species levels, *Porphyromonas* was significantly enriched in the DC-L group, while *Streptococcus parasanguinis_clade_411* was notably enriched in the DC-H group. These results indicate that in the caries group, although differences in sweet consumption frequency did not change the overall microbiome structure, the differences at the specific genus/species levels might reflect the dominant microbial traits under varying sweet exposure conditions. To further identify the specific differential microbiomes based on varying sweet consumption frequencies in the caries group, we applied the LEfSe method (LDA score >3.0, FDR < 0.05) to compare the microbiome composition between the high sweet consumption frequency group (DC-H) and the low sweet consumption frequency group (DC-L). The results identified 7 significant differential taxonomic units: In the DC-H group, the species enriched included *Prevotella salivae* (*s_salivae*), *Veillonella atypica* (*s_atypica*), *Streptococcus parasanguinis clade 411* (*s_parasanguinis_clade_411*), *Rothia dentocariosa* (*s_dentocariosa*), and *Veillonella dispar* (*s_dispar*). These bacteria are closely related to sugar metabolism, acid production, and the progression of caries. The species enriched in the DC-L group included *Haemophilus* sp. *HMT* 908 (*s_sp_HMT_908*) and *Porphyromonas gingivalis* (*g_Porphyromonas*; *s_gingivalis*). They are typically related to the maintenance of oral health or periodontal-associated microbiota. In summary, the results indicate that in the caries group, high sweet consumption frequency significantly changes the salivary microbiome structure, promoting the enrichment of acid-producing and acid-tolerant bacteria; while low sweet consumption frequency helps maintain microbiota traits associated with periodontal health.

### Comparison of salivary microbes between DF-L and DF-H

**Figure 4:**
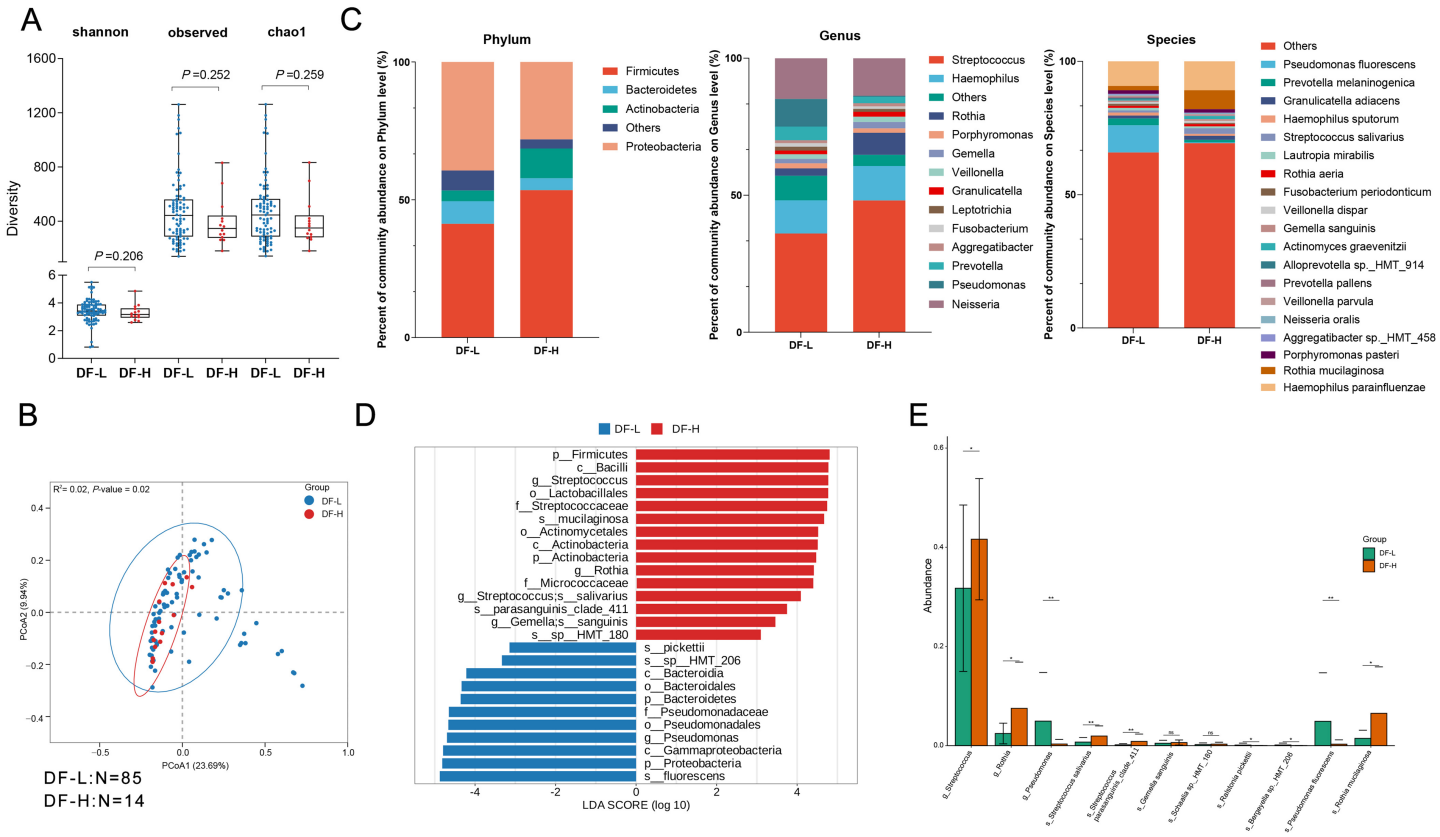
Comparison of oral microbiota diversity and composition between DF-L and DF-H groups. **(A)** Alpha diversity comparison showing no significant differences between the DF-L and DF-H groups for Shannon, Observed, and Chao1 indices (*p* > 0.05, Wilcoxon test). **(B)** PCoA (Principal Coordinates Analysis) plot based on Bray–Curtis distances, illustrating the microbial community structure between the two groups. Each point represents a sample, and colors differentiate the groups. The *R*^2^ value is 0.02, and the *p*-value is 0.02, indicating a significant separation between the groups. **(C)** Average relative proportions of the main families, genera, and species in the two groups. Bar charts show the relative abundance of microbial taxa at different taxonomic levels. **(D)** LEfSe analysis (LDA > 3) identifying key bacterial taxa with significant differences in abundance (from phylum to species level) between the DF-L and DF-H groups. Only significant taxa with LDA scores greater than 3 are displayed. **(E)** Bar chart showing the comparison of a specific microbial taxon identified by LEfSe analysis (LDA > 3) between the two groups, illustrating the significant difference in microbial abundance between DF-L and DF-H. DF-H (*N* = 14): the high sweet consumption frequency in the dental fluorosis group; DF-L (*N* = 85): the low sweet consumption frequency in the dental fluorosis group.

In the fluorosis group without caries (DF group), we compared the salivary microbiome composition between the high sweet consumption frequency (DF-H) and low sweet consumption frequency (DF-L) groups. α-diversity analysis showed that at the ASV level, there were no significant differences in Shannon, Simpson, and Chao1 indices between DF-L and DF-H (Figure 4A), suggesting that differences in sweet consumption frequency did not alter the richness or evenness of the community. β-diversity analysis based on Bray–Curtis distance showed separation of DF-L and DF-H samples in coordinate space (Figure 4B), and PERMANOVA testing suggested that the difference was statistically significant (*R*^2^ = 0.02, *p* = 0.02), indicating detectable differences in the overall community structure between the two groups. Community abundance comparisons showed (Figure 4C): At the phylum level, DF-L was enriched in *Proteobacteria* and *Bacteroidetes*, while DF-H was enriched in *Firmicutes* and *Actinobacteria*. At the genus level, *Streptococcus* and *Rothia* were significantly more abundant in DF-H, while *Pseudomonas* was enriched in DF-L. At the species level, *Rothia mucilaginosa* and *Streptococcus sali*var*ius* were enriched in DF-H, while *Pseudomonas fluorescens* was enriched in DF-L. These results suggest that the DF group exhibits notable differences in dominant microbiota profiles under varying sweet consumption frequencies. LEfSe analysis (LDA score > 3.0, FDR < 0.05) further confirmed this trend (Figure 4D): The DF-H group was enriched in *Streptococcaceae* (*S. salivarius, S. parasanguinis clade 411*) and *Rothia* (*R. mucilaginosa*), while the DF-L group was enriched in *Pseudomonas* (*P. fluorescens*). In conclusion, in the fluorosis group without caries, high sweet consumption frequency did not lower overall diversity but significantly altered the community structure, with the enrichment of potential cariogenic or acid-producing bacteria like *Streptococcus* and *Rothia*. Low sweet consumption frequency was dominated by microbiota like *Pseudomonas fluorescens*, which may play a role in maintaining oral homeostasis. Therefore, even without caries, individuals with fluorosis may be at risk of their oral microbiome shifting toward a cariogenic direction under a high-sugar diet.

### Microbial differences in HC-H, DC-H and DF-H group

**Figure 5:**
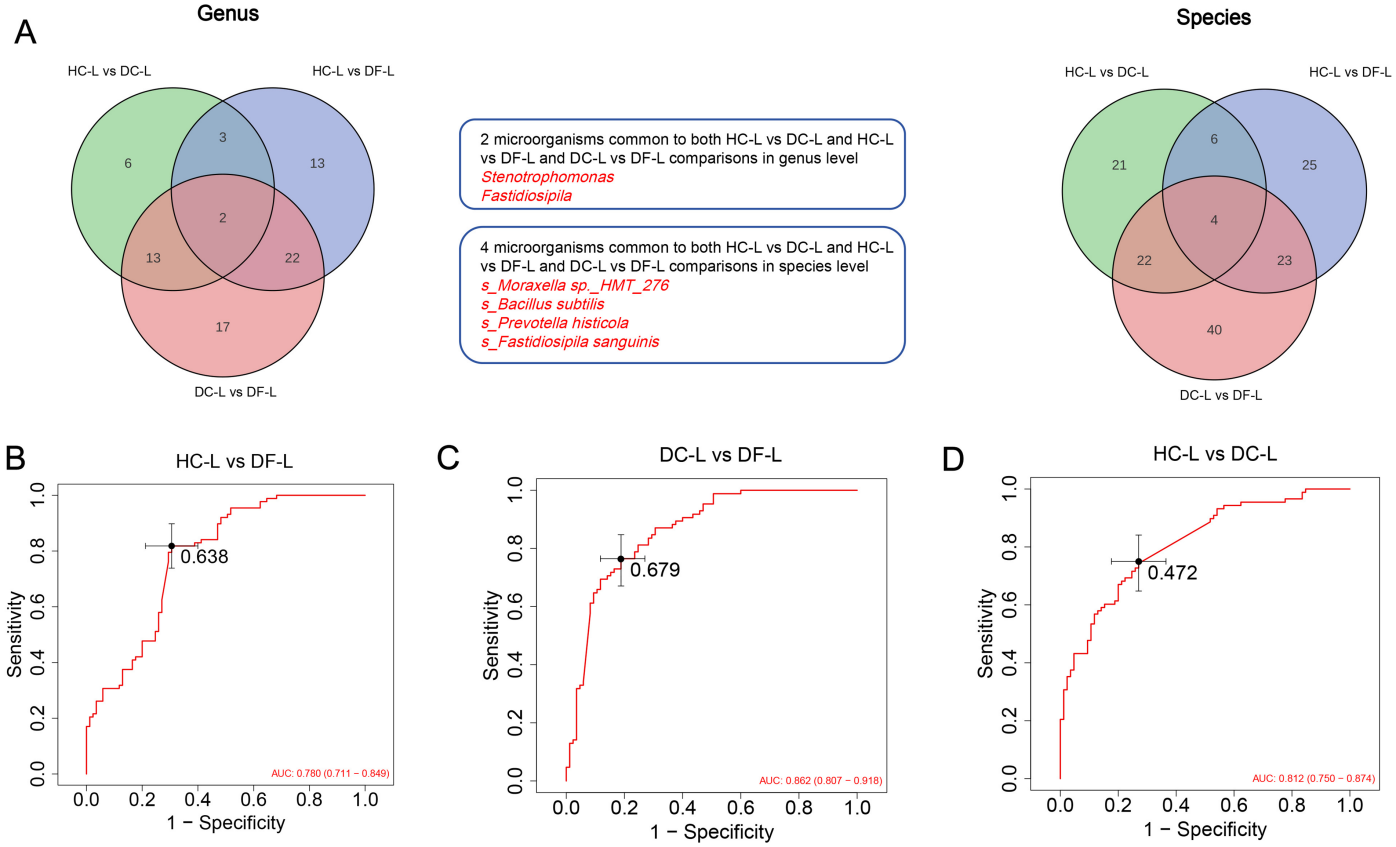
**(A)** Venn diagram: used to compare the differential microorganisms at the genus and species level among the three groups of samples, namely HC-L, DC-L, DF-L; 2 microorganisms common to both HC-L vs. DC-L and HC-L vs. DF-L and DC-L vs. DF-L comparisons in genus level *Stenotrophomonas*, *Fastidiosipila*. 4 microorganisms common to both HC-L vs. DC-L and HC-L vs. DF-L and DC-L vs. DF-L comparisons in species level *s_Moraxella* sp.*_HMT_276, s_Bacillus subtilis, s_Prevotella histicola, s_Fastidiosipila sanguinis*. **(B–D)** ROC analysis for the 4 microorganisms markers discriminating HC-L vs. DC-L and HC-L vs. DF-L and DC-L vs. DF-L. DC-L (*N* = 85): The low sweet consumption frequency in the dental caries group; HC-L (*N* = 88): The low sweet consumption frequency in the healthy control group; DF-L (*N* = 85): The low sweet consumption frequency in the dental fluorosis group.

Analysis of oral microbiota diversity and composition revealed no significant differences in Shannon, Observed, or Chao1 indices between the low-score (HC-L, DC-L, DF-L) or high-score (HC-H, DC-H, DF-H) groups (*p* > 0.05, Wilcoxon test). Principal coordinates analysis based on Bray–Curtis distances showed no clear separation in microbial community structure among groups. Furthermore, the relative abundances of major genera and species at different taxonomic levels did not differ significantly between groups (Supplementary Figure S1). To explore the oral microbiome response patterns under the strong influence of high-frequency sweet food, we integrated the differentially expressed microorganisms at the genus and species levels (selection criteria: FC > 2 or < 0.5, FDR < 0.05) into a Venn diagram for comparison (Figures 5A,B). The analysis identified a total of seven intersecting sets. Notably, at both the genus and species levels, a unique core bacterium, *Ralstonia*, was observed to show differential abundance across all three group comparisons, with abundance ordered as DC-H (caries) > HC-H (healthy) > DF-H (fluorosis). This result indicates that *Ralstonia* may have a close association with the occurrence of caries in a high-sugar environment. At the species level, we further identified 8 differential bacteria between DC-H and DF-H (e.g., *s_Parvimonas micra*, *s_Streptococcus mutans*, *s_Prevotella melaninogenica*) and 7 DC-specific bacteria (e.g., *s_Lachnoanaerobaculum orale*, *s_Veillonella dispar*, *s_Prevotella oris*, *s_Prevotella denticola*). These results indicate that in a high-sugar environment, caries-associated dominant microbiota are enriched, forming a typical caries-dominated core microbiota.

### Microbial differences in HC-L, DC-L and DF-L group

**Figure 6:**
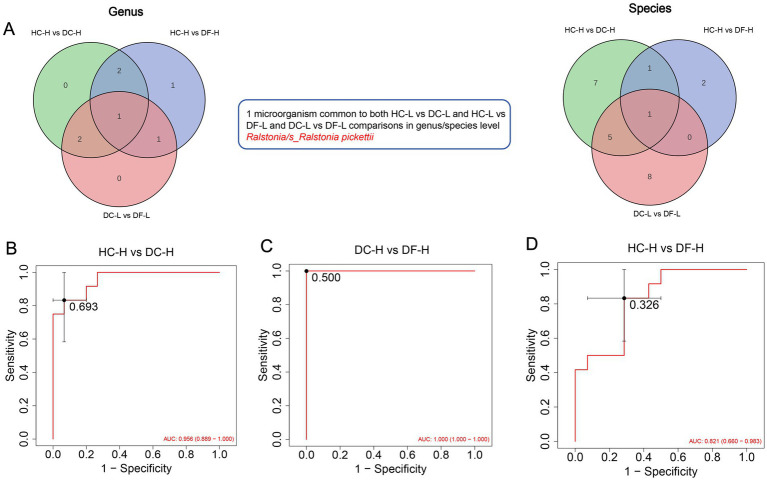
**(A)** Venn diagram: used to compare the differential microorganisms at the genus and species level among the three groups of samples, namely HC-H, DC-H, DF-H; 1 microorganism common to both HC-H vs. DC-H and HC-H vs. DF-H and DC-H vs. DF-H comparisons in genus/species level. *Ralstonia/s_Ralstonia pickettii*. **(B–D)** Receiver operating characteristic (ROC) curves of the 1 microorganisms markers discriminating HC-H vs. DC-H and HC-H vs. DF-H and DC-H vs. DF-H. DC-H (*N* = 15): the high sweet consumption frequency in the dental caries group; HC-H (*N* = 12): the high sweet consumption frequency in the healthy control group; DF-H (*N* = 14): the high sweet consumption frequency in the dental fluorosis group.

In contrast, under low-frequency sweet food conditions, we also combined the differential microbiota at the genus and species levels into a Venn diagram (Figures 6A,B). The analysis revealed that the three groups showed significant differences in their microbiome characteristics. First, the core bacteria showing differences in all three group comparisons included *Stenotrophomonas* and *Fastidiosipila*. The abundance of *Stenotrophomonas* was in the order of HC-L > DF-L > DC-L, indicating it may be a dominant bacterium in health, helping to maintain oral ecological stability and suppress the expansion of cariogenic microbiota in a low-sugar environment. Its reduction could be associated with microbiota imbalance in the caries group. The abundance of *Fastidiosipila* was in the order of DF-L ≫ HC-L > DC-L, showing significant enrichment in the fluorosis group. This result suggests that it may represent a fluoride-adapted microbiota, with its dominance reflecting the selective pressure of fluoride on the oral microbiome and potentially granting the fluorosis group a distinct ecological niche. Furthermore, pairwise comparisons further revealed community differences: 17 differential bacteria between DC-L and DF-L, suggesting that even in low-sugar conditions, distinct microbial differentiation exists between caries and fluorosis. These microbial groups may be key microorganisms responsible for the phenotypic differences between the two diseases, reflecting the fundamental differences in ecological adaptation between caries and fluoride exposure.13 differential bacteria between HC-L and DF-L, indicating that the fluorosis group is different not only from the caries group but also from healthy individuals. This suggests that the microbiota in the fluorosis group is not simply “in between health and caries,” but rather forms an independent microbial ecological pattern.

To evaluate the biological relevance of between-group differences and assess the reliability of our statistical conclusions, we quantified the magnitude of these differences by calculating Hedges’ *g* effect size along with its 95% confidence interval. The results indicated only minimal effects, with all g values below 0.2 and confidence intervals encompassing zero. Consistently, the β-diversity analyses for HC-L vs. HC-H and DC-L vs. DC-H groups revealed exceptionally low *R*^2^ values (< 2%). Those may indicate that the effect of this factor on α and β-diversity is weak, and its potential ecological role needs to be further evaluated by combining functional data, network analysis, or multi-model inference. This further supports the statistical conclusion that no substantial differences exist between these group pairs.

Although a statistically significant difference in β-diversity was observed between DF-L and DF-H groups based on sweet food consumption frequency (*R*^2^ = 0.02, *p* = 0.02), several considerations suggest limited biological importance. As emphasized by Nakagawa and Cuthill (2007), biological interpretation should prioritize practical effect magnitude over statistical significance alone, since minimal effects may achieve significance in large samples yet lack ecological relevance. Additionally, methodological work by Caporaso and colleagues highlights that microbial community data typically exhibit high baseline variation (high within-group heterogeneity) (He et al., 2015). Reliable detection (e.g., with >80% statistical power) of effects explaining less than 2% of variance generally requires sample sizes exceeding 200, often reaching 500 or more—far beyond the sample size available for the DF-L and DF-H groups in this study. Therefore, despite the statistical significance, we consider the biological relevance of this difference to be limited, as the grouping factor explains only approximately 2% of community variation and no clear separation is visible in PCoA ordination. These findings suggest that microbial community variation is primarily driven by other, unmeasured factors.

## Discussion

Previous studies have established that the frequency of sweet food and sugary beverage consumption significantly alters the salivary microbiota in individuals with typical plaque-related diseases, such as dental caries, and healthy individuals (Fan et al., 2024; Lommi et al., 2022; Tang et al., 2024). Microbiomic evidence suggests that these differences may be related to variations in glycosidase activity, which influences how colonizing microbiota respond to sugar metabolism (Esberg et al., 2020). However, the impact of sugar intake on non-plaque-related diseases, such as dental fluorosis, as well as the differential effects across healthy individuals, caries patients, and those with dental fluorosis, remain insufficiently explored. In this study, we compared the salivary microbial communities across three distinct groups (healthy individuals, dental caries patients, and dental fluorosis patients) under different sweet food consumption conditions. While no significant differences in α-diversity were observed between the groups, distinct structural changes in the microbiota were noted. For instance, high sweet food consumption in the dental fluorosis group led to an enrichment of *Rothia mucilaginosa*, while the caries group exhibited an overrepresentation of acid-producing bacteria such as *Prevotella salivae*, *Veillonella atypica*, *Streptococcus parasanguinis*, and *Rothia dentocariosa*. Conversely, under low sweet food intake, the dental fluorosis group was characterized by *Pseudomonas fluorescens*, and the caries group by *Porphyromonas gingivalis*. These findings underscore the differential microbiological effects of sweet food consumption in various oral health conditions and provide new evidence for the complex interplay between diet, oral diseases, and the microbiome.

For this study, dental fluorosis was selected as a representative non-plaque-related oral disease. Bengbu, located in northern Anhui Province, has a distinct regional population. Liu et al. reported a 47% prevalence of dental fluorosis among 957 university students in Bengbu, closely mirroring the 50% prevalence in the broader population (Liu et al., 2023). This high prevalence not only reflects the widespread nature of dental fluorosis in the region but also enhances the external validity and representativeness of our findings. To ensure experimental feasibility and sample consistency, we chose university students, a group with more uniform lifestyle factors (e.g., weight, smoking, and alcohol consumption), which reduces potential confounding variables (Nazareno et al., 2020). Additionally, the generally good oral health of university students, with low rates of periodontitis, provides a stable context for studying the relationship between diet and the oral microbiome. In microbiome analysis, different salivary sample collection methods can significantly impact community composition and diversity (Bang et al., 2023). Previous studies have assessed the effects of passive drooling versus rinsing methods, as well as different saliva preservation techniques, highlighting the importance of sampling standardization (Omori et al., 2020). In this study, samples were collected after brushing and rinsing, following an overnight fast, under consistent preservation conditions to minimize confounding factors. As noted in the introduction, oral microbiota can be categorized into those attached to biofilms and those suspended in saliva. Post-rinse saliva samples are more effective in capturing planktonic microorganisms, which are more mobile and can be transmitted between different areas of the mouth and individuals through saliva flow or droplets. Moreover, saliva, as a key vector for infectious diseases like caries, contains planktonic microorganisms that play a crucial role in disease transmission. Thus, incorporating post-rinse saliva samples broadens the scope of oral microbiome studies, offering new insights into the role of planktonic microorganisms in the occurrence and transmission of oral diseases.

This study found that while the frequency of sweet food intake did not significantly affect α-diversity levels across the three disease groups. Moreover, this study revealed that the grouping factor had only a minimal effect on the overall microbial community structure (β-diversity, *R*^2^ < 0.02). However, further differential abundance analysis of individual taxa demonstrated that, despite the limited overall dissimilarity, several key bacterial groups exhibited statistically significant abundance differences between the conditions. In the high sweet food intake group (DF-H), *Rothia* and *Rothia mucilaginosa* were notably enriched. Previous studies have similarly reported increased abundance of *R. mucilaginosa* in the saliva of children with early childhood caries and individuals with severe dental fluorosis (Grier et al., 2020; Wang et al., 2021). *In vitro* experiments have also shown a dramatic increase in *R. mucilaginosa* abundance following sodium fluoride treatment of oral biofilms (López-López and Mira, 2020), aligning with our findings. This suggests that fluoride-related environments and sugar metabolism may synergistically promote the proliferation of *R. mucilaginosa*. In contrast, the low sweet food intake group (DF-L) was characterized by an enrichment of *Pseudomonas fluorescens*. Previous research has shown that *P. fluorescens* secretes *L-asparaginase*, which inhibits the formation of cariogenic biofilms (Muslim et al., 2024), and has been found to significantly enrich in the black tooth plaque (BTS) of caries-free individuals, with a positive correlation to the number of discolored teeth (Zhang et al., 2022). Studies have also demonstrated that individuals with BTS generally have a lower caries incidence (Chen et al., 2021), suggesting that *P. fluorescens* may play a protective role under certain environmental conditions. In contrast to studies that focus solely on the amplification of cariogenic bacteria in high-sugar environments, this study not only confirms the well-established association between sweet food intake and the enrichment of *Rothia* but also provides the first report of *Pseudomonas fluorescens* being linked to low-sugar exposure in the dental fluorosis (DF) group. This finding introduces a bidirectional model, suggesting that “high sugar promotes the proliferation of cariogenic bacteria, while low sugar maintains potentially protective microbial populations.” These results imply that, even in the absence of caries, individuals with dental fluorosis may face an increased risk of their oral microbiota shifting toward a cariogenic profile under high-sugar dietary conditions.

In the DC-H group, we observed significant enrichment of *Prevotella salivae*, *Veillonella atypica*, *Streptococcus parasanguinis evolutionary branch 411*, *Rothia dentocariosa*, and *Veillonella dispar*. These microorganisms are generally known for their strong sugar metabolism and acid production capabilities, which aligns closely with the findings of Lommi et al. (2022), who reported higher abundance of *Streptococcus*, *Prevotella*, *Veillonella*, and *Selenomonas* in the high-sugar intake caries population compared to the low-sugar intake group. This further underscores the significant role of high sweet food consumption in driving the imbalance of caries-associated microbiota. However, Lommi et al. (2022) employed the traditional OTU clustering method and the SILVA database. SILVA is a comprehensive rRNA gene sequence database primarily containing 16S/18S rRNA gene sequences from bacteria, archaea, and eukaryotes (Campos et al., 2022). In contrast, our study utilized both the ASV method for sequence analysis and the eHOMD database for taxonomic annotation. ASV, a more precise molecular marker approach, also known as the exact sequence variant (ESV) method, employs denoising algorithms such as DADA2 and Deblur to resolve true variant sequences within each sample, thus avoiding the information loss associated with OTU clustering due to similarity thresholds (97%). This approach allows for a more comprehensive and accurate depiction of the oral microbiota composition and potential functions (Plessis et al., 2023; Mat-Hussin et al., 2024). The DC-L group, on the other hand, was characterized by the enrichment of *Porphyromonas gingivalis*. While *Porphyromonas gingivalis* is widely recognized as a periodontal pathogen (Mysak et al., 2014), its relative abundance in the low-sweet food condition suggests that, in sugar-limited environments, it may occupy a specific ecological niche. The risk of periodontitis is relatively high in the population with caries and the frequency of sweet food is high.

In this study, we provide the first evidence of differential abundance changes in the core bacterium *Ralstonia* driven by high-frequency sweet food consumption. Our findings show that *Ralstonia* abundance is significantly higher in the dental caries (DC) group compared to both the healthy (5-fold) and dental fluorosis (50-fold) groups. This novel discovery suggests that *Ralstonia* may play an important role in the development of dental caries. *Ralstonia*, typically considered a Gram-negative opportunistic pathogen, is known for its resilience in hospital environments, where it can survive in various solutions. In immunocompromised individuals, contamination of these solutions may lead to bacteremia (Baker et al., 2022; Saunders et al., 2024), such as catheter-associated bloodstream infections caused by *Ralstonia pickettii* from contaminated saline (Bedir Demirdag et al., 2022). Furthermore, studies have shown that *Ralstonia pickettii* is linked to higher mortality rates in patients undergoing allogeneic hematopoietic stem cell transplantation (allo-HSCT) when its presence in the oral microbiome is detected on the day of transplantation (Oku et al., 2020). Additionally, *Ralstonia pickettii* has been found in higher proportions in the saliva of periodontal disease patients, and its abundance decreases when blood glucose is effectively controlled in patients with type 2 diabetes and periodontal disease (Sun et al., 2020).

In contrast, under low-sugar conditions, we observed the enrichment of *Stenotrophomonas* and *Fastidiosipila*, both core bacteria. *Stenotrophomonas* was 90 times more abundant in the DC-L group compared to the HC-L group and 15 times more abundant in the DF-L group. *Fastidiosipila*, on the other hand, was enriched 234 times in the DF-L group compared to the DC-L group, and 80 times more abundant in the DF-L group than in the HC-L group. These patterns suggest a close association between these bacteria and either health or dental fluorosis. Notably, *Stenotrophomonas* showed higher abundance in the healthy group than in either the dental fluorosis or caries groups, although its presence was relatively low. This genus includes a few pathogenic strains, but its primary role seems to be in maintaining ecological balance (Ryan et al., 2009). On the other hand, the enrichment of *Fastidiosipila* in the dental fluorosis group indicates selective pressure exerted by fluoride on the oral microbiota. *Fastidiosipila sanguinis*, an anaerobic Gram-negative bacterium newly identified in 2005 (Falsen et al., 2005), is typically found in the blood and has the potential to cause bacteremia (Beauruelle et al., 2014). The exact role of this bacterium remains unclear, but due to its sensitivity to oxygen, *Fastidiosipila sanguinis* likely thrives in the anaerobic environments of the oral cavity and may interact with other pathogens, influencing oral health.

Despite the innovative aspects of this study, several limitations remain, which should be addressed and improved upon in future research. First, given the cross-sectional design used in this study, future research should consider employing a longitudinal design to track individual changes in the microbiota and oral health status over time. Additionally, incorporating dynamic data analysis, such as time-series analysis, could provide a more detailed understanding of the long-term evolution of the oral microbiome. Second, regarding the limitations of sample size, future studies should expand the sample size through multicenter collaborations, especially by including representative samples from different regions and populations, which would enhance the external generalizability of the results. Furthermore, adopting multi-omics approaches-combining genomics, metabolomics, proteomics, and other layers of data-could offer a more comprehensive insight into the mechanisms by which sweet food intake frequency impacts the oral microbiota, thereby deepening and broadening the scope of the research. Lastly, future studies should focus on more refined disease classifications, particularly the microbiological differences between dental caries and dental fluorosis. Combining *in vitro* experiments, such as culturing clinically isolated strains at different sugar concentrations, or animal models for preliminary validation, could help explore potential cross-effects and dynamic shifts in microbial communities. This would not only aid in further understanding the ecological characteristics of different oral diseases but also provide scientific evidence for the development of personalized oral health intervention strategies.

## Conclusion

In conclusion, while this study has certain limitations, it provides valuable insights into the relationship between high-frequency sweet food consumption and the oral microbiome. Our detailed analysis revealed distinct and significant differences in the salivary microbiota profiles among individuals with different oral health statuses—namely, healthy, dental caries, and dental fluorosis. Specifically, we identified that a high-frequency sweet food intake is associated with a marked reduction in microbial diversity and an enrichment of cariogenic bacteria in the caries group, whereas a different, potentially fluoride-resistant microbiota consortium was observed in the fluorosis group. These findings not only delineate the specific microbial shifts under different clinical conditions but also offer a novel theoretical perspective by suggesting that the host’s sweet preference acts as a key modulator, driving the microbiome toward divergent disease-associated states. This underscores the importance of dietary counseling in the prevention of oral diseases.

## Data Availability

The datasets presented in this study can be found in online repositories. The names of the repository/repositories and accession number(s) can be found below: https://www.ncbi.nlm.nih.gov/, PRJNA865814; PRJNA822496.

## Footnotes

1
http://ccb.jhu.edu/software/FLASH/

2
https://github.com/OpenGene/fastp

3
https://github.com/torognes/vsearch/

4
https://ftp.ncbi.nlm.nih.gov/pub/taxonomy/taxdump.tar.gz

## Supplementary material

SUPPLEMENTARY FIGURE S1 Comparison of oral microbiota diversity and composition between HC and DC and DF groups. **(A)** Alpha diversity comparison showing no significant differences between the HC-L and DC-L and DF-L groups for Shannon, Observed, and Chao1 indices (*p* > 0.05, Wilcoxon test). PCoA (Principal Coordinates Analysis) plot based on Bray-Curtis distances, illustrating the microbial community structure between the HC-L and DC-L and DF-L groups. Average relative proportions of the main genera, and species in the three groups. Bar charts show the relative abundance of microbial taxa at different taxonomic levels. **(B)** Alpha diversity comparison showing no significant differences between the HC-H and DC-H and DF-H groups for Shannon, Observed, and Chao1 indices (*p* > 0.05, Wilcoxon test). PCoA (Principal Coordinates Analysis) plot based on Bray-Curtis distances, illustrating the microbial community structure between the HC-H and DC-H and DF-H groups. Average relative proportions of the main genera, and species in the three groups. Bar charts show the relative abundance of microbial taxa at different taxonomic levels. DC-H: the high sweet consumption frequency in the dental caries group; DC-L: The low sweet consumption frequency in the dental caries group; HC-H: the high sweet consumption frequency in the healthy control group; HC-L: The low sweet consumption frequency in the healthy control group; DF-H: the high sweet consumption frequency in the dental fluorosis group; DF-L: The low sweet consumption frequency in the dental fluorosis group.
