# Supplementary figures and images for: Host sweet preference modulates the salivary microbiome and its divergent associations with plaque-associated and non-plaque-related oral diseases
Source: Front Microbiol. 2025 Dec 5;16:1732083. doi: 10.3389/fmicb.2025.1732083 (PMC12715807; doi:10.3389/fmicb.2025.1732083)

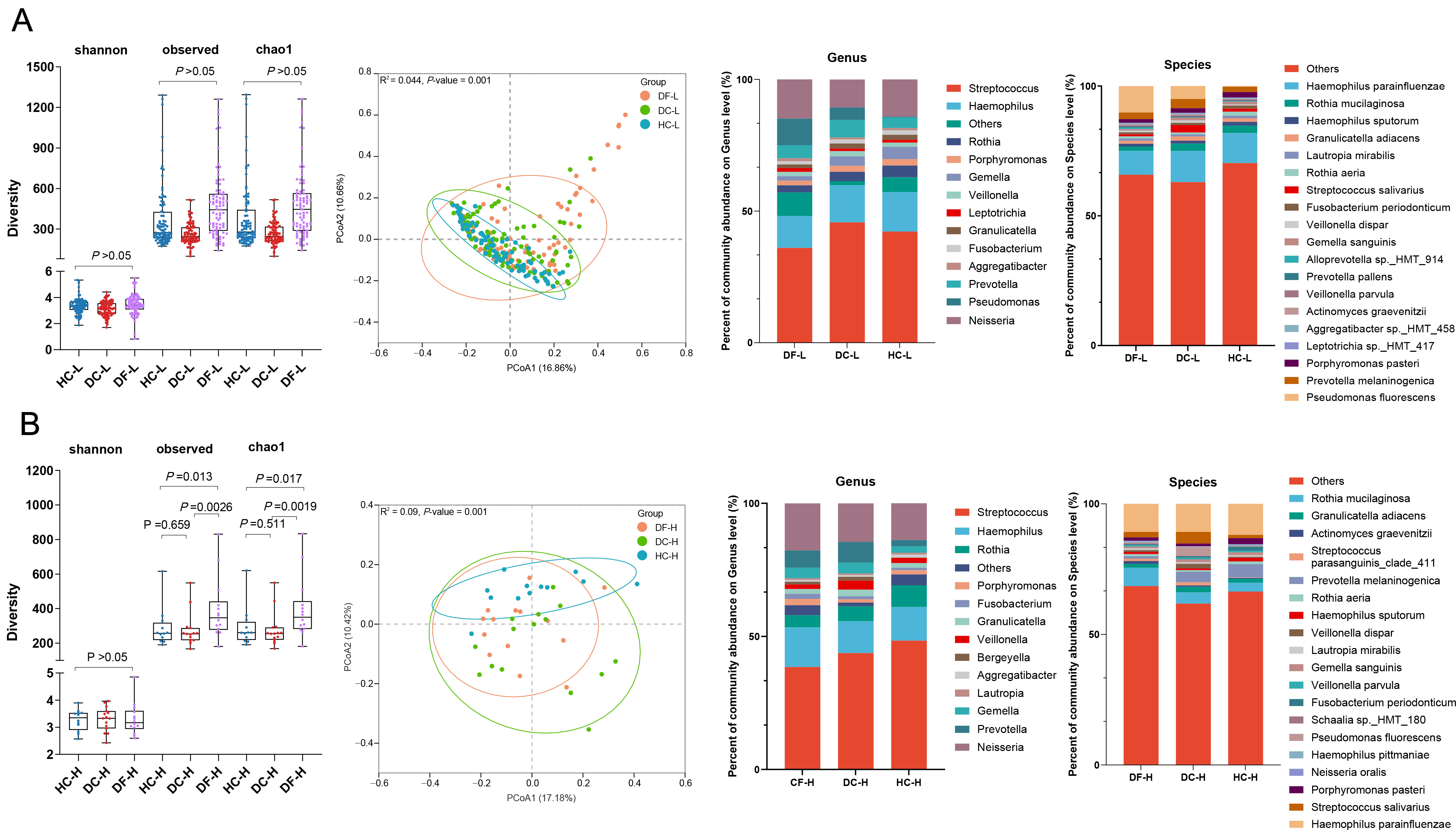

Supplement: SUPPLEMENTARY FIGURE S1 — Comparison of oral microbiota diversity and composition between HC and DC and DF groups. (A) Alpha diversity comparison showing no significant differences between the HC-L and DC-L and DF-L groups for Shannon, Observed, and Chao1 indices (p > 0.05, Wilcoxon test). PCoA (Principal Coordinates Analysis) plot based on Bray-Curtis distances, illustrating the microbial community structure between the HC-L and DC-L and DF-L groups. Average relative proportions of the main genera, and species in the three groups. Bar charts show the relative abundance of microbial taxa at different taxonomic levels. (B) Alpha diversity comparison showing no significant differences between the HC-H and DC-H and DF-H groups for Shannon, Observed, and Chao1 indices (p > 0.05, Wilcoxon test). PCoA (Principal Coordinates Analysis) plot based on Bray-Curtis distances, illustrating the microbial community structure between the HC-H and DC-H and DF-H groups. Average relative proportions of the main genera, and species in the three groups. Bar charts show the relative abundance of microbial taxa at different taxonomic levels. DC-H: the high sweet consumption frequency in the dental caries group; DC-L: The low sweet consumption frequency in the dental caries group; HC-H: the high sweet consumption frequency in the healthy control group; HC-L: The low sweet consumption frequency in the healthy control group; DF-H: the high sweet consumption frequency in the dental fluorosis group; DF-L: The low sweet consumption frequency in the dental fluorosis group. [file Image_1.jpeg]
